# Genetic structure and demographic history of house mice in western Europe inferred using whole-genome sequences

**DOI:** 10.1098/rspb.2024.2709

**Published:** 2025-04-16

**Authors:** Kennedy Agwamba, Lydia Smith, Sofia I. Gabriel, Jeremy B. Searle, Michael W. Nachman

**Affiliations:** ^1^ Museum of Vertebrate Zoology, University of California Berkeley, Berkeley, CA, USA; ^2^ Center for Computational Biology, University of California Berkeley, Berkeley, CA, USA; ^3^ Department of Animal Biology, Faculty of Sciences, University of Lisbon, Centre for Ecology Evolution and Environmental Changes, Lisbon, Portugal; ^4^ Department of Ecology and Evolutionary Biology, Cornell University, Ithaca, NY, USA; ^5^ Integrative Biology, University of California Berkeley, Berkeley, CA, USA

**Keywords:** western Europe, population genetics, house mouse, demographic history, whole-genome sequences, commensal

## Abstract

The western house mouse, *Mus musculus domesticus*, is a human commensal and an outstanding model organism for studying a wide variety of traits and diseases. However, we have few genomic resources for wild mice and only a rudimentary understanding of the demographic history of house mice in Europe. Here, we sequenced 59 whole genomes of mice collected from England, Scotland, Wales, Guernsey, northern France, Italy, Portugal and Spain. We combined this dataset with 24 previously published sequences from southern France, Germany and Iran and compared patterns of population structure and inferred demographic parameters for house mice in western Europe to patterns seen in humans. Principal component and phylogenetic analyses identified three genetic clusters in western European mice. Admixture and *f*-branch statistics identified historical gene flow between these genetic clusters. Demographic analyses suggest a shared history of population bottlenecks prior to 20 000 years ago. Estimated divergence times between populations of house mice from western Europe ranged from 1500 to 5500 years ago, in general agreement with the zooarchaeological record. These results correspond well with key aspects of contemporary human population structure and the history of migration in western Europe, highlighting the commensal relationship of this important genetic model.

## 1. Introduction

Detailing the history of demographic and evolutionary processes that have shaped modern populations is a fundamental goal in evolutionary and ecological studies. Species that share a close association may influence each other’s population dynamics and movements (e.g. [1]). One kind of association that has become particularly important during the Anthropocene involves humans and the animals that live in close association with them. In particular, the distribution and diversification of human commensal species can be greatly influenced by the movements of human populations [2–10].

The house mouse (*Mus musculus*) is a human commensal, a premier mammalian model organism for biological research and an invasive pest responsible for costly damages to human infrastructure and food reserves [11]. House mice consist of three major subspecies which diverged from an ancestral population in the northern Indo-Pakistani subcontinent and neighbouring regions roughly 200 000−500 000 years ago [12–19]: *Mus musculus castaneus* in Southeast Asia*, Mus musculus musculus* in eastern Europe and northern Asia, and *M. m. domesticus* in the Middle East and western Europe. In association with humans, *M. m. domesticus* extended its geographic range to Africa, Australia and the Americas during the last several centuries, presumably as a consequence of European colonialism [17,20–27]. Therefore, an understanding of the population history and structure of house mice in western Europe lays the foundations for identifying the source populations of house mice that have been spread to other regions of the world by Europeans.

Much of our current understanding of the history of house mice in western Europe comes from the zooarchaeological record [9,28,29]. This record suggests that human–house mouse commensalism dates to between 12 000 and 15 000 years ago [9,28,30]. Some authors have argued that house mice have since become an anthrodependent species [5]. The introduction of early farming practices is thought to have enabled the cohabitation of humans and house mice in the Near East during the Neolithic. Despite notable human migration from the Near East to western Europe during the Neolithic, the spread of house mice was limited to movement from the Near East to the eastern Mediterranean and Anatolia around this time. Following the Neolithic, the next notable period of diffusion occurred in the Late Bronze and Early Iron Age, with rapid spread across the western Mediterranean. Increased maritime transport, together with the presence of more substantial human settlements in western Europe, is suspected to have played a major role [9,28]. The first direct evidence of house mice found on ships was obtained from the shipwreck of a Late Bronze Age vessel found off the coast of Turkey [29]. The most recent zooarchaeological survey dates the arrival of *M. m. domesticus* in southern Europe around 4000 years ago and in western and northern Europe around 3000 years ago [9].

A small number of studies have used modest sets of genetic data to analyse present-day population structure and reconstruct the diffusion history of house mice in western Europe. For example, previous studies have identified ‘Mediterranean-like’ and ‘northern European-like’ clusters based on mitochondrial DNA or microarrays using pre-ascertained single nucleotide polymorphisms [17,31]. A study using ancient mtDNA from the British Isles provided evidence for a Late Bronze to Iron Age colonization [26]. Researchers have then relied on these limited groupings of house mice to propose dispersal routes within western Europe in concordance with the zooarchaeological records [9,22,23,28,31]. Whole-genome sequences, in contrast, provide an opportunity to more completely survey variation across the genome and exploit more powerful computational and statistical inference procedures [32–34]. In particular, they provide data with which to estimate evolutionary relationships, patterns of historic gene flow, divergence times and ancestral population sizes.

Here, we gather 83 whole-genome sequences of wild-caught *M. m. domesticus*, including 59 new whole-genome sequences of mice from England, Scotland, Wales, Guernsey (an island in the English Channel just off the coast of northern France), northern France, Italy, Portugal and Spain. We use these data to infer the genetic structure of house mice in western Europe, the phylogenetic relationships of populations, the amount of gene flow between populations and historical changes in population size. We provide estimates of divergence times between lineages to update previous models of house mouse history in western Europe. We then compare our findings with similar studies in humans to assess the concordance between these species. For example, human populations in western Europe exhibit isolation by distance (IBD) [35,36]; do we see similar patterns in mice over the same geographic scale? Does the genetic structure of mice correspond to geopolitical boundaries found in modern or past human populations? If mice are constrained largely to dispersal by humans, we would expect strong concordance. If, on the other hand, mice can disperse long distances independently of human movements, we would expect little concordance. The latter pattern is characteristic of some commensal species such as starlings, which quickly spread after their initial introduction in North America [37,38]. We find that mice in western Europe show a strong signal of IBD, similar to humans, and that mice form genetic clusters that generally match human genetic clusters. In some cases, house mice exhibit genetic discontinuities that coincide with current political boundaries over short geographic distances, such as between Portugal and Spain. In other cases, house mice exhibit genetic patterns more aligned to historical geopolitical associations, such as the Roman Empire. In general, the inferred timing of divergence between mouse clusters is found to be consistent with human migration history.

## 2. Material and methods

### (a) Sample description

We gathered 83 whole-genome sequences of *M. m. domesticus* sampled from western Europe and Iran. This included 24 samples from a previously published dataset representing populations from southern France, Germany and Iran [39]. The remaining 59 samples are newly sequenced genomes of wild-caught mice collected in northern France, Italy, northeast Spain (Catalonia), southwest Spain, Portugal, England, Wales, Scotland and Guernsey. Mice from these locations were chosen from existing specimen collections, in part to capture the probable geographic regions from which mice were spread by Europeans to other parts of the world. Specimen ID, sex, collecting locality, and year of capture for each individual are given in electronic supplementary material, tables S1 and S2. Detailed molecular methods are described in electronic supplementary material, text S1.

### (b) Single nucleotide polymorphism discovery

Sequencing reads were processed using a standardized pipeline, following the general procedures and parameters outlined in Harr *et al.* [39] to produce sequence alignments. Raw Illumina sequencing reads were first cleaned and preprocessed using FastP [40], then aligned to the mouse reference genome (GRCm38/mm10, RefSeq: GCF_000001635.20) using BWA-MEM [41]. Further bioinformatic details together with the pipelines used for processing raw sequences are provided in the electronic supplementary materials. To compare genetic variation in mice and humans, the Human Origins dataset was downloaded from a public online repository (https://reich.hms.harvard.edu/sites/reich.hms.harvard.edu/files/inline-files/NearEastPublic.tar.gz).

Alignment statistics were generated using samtools [42,43]. Specifically, total reads passing quality control and the percent of mapped reads were computed using ‘samtools flagstat’, and average per-base autosomal and sex coverage were computed using ‘samtools depth’. Given the modest coverage of 7−20× per individual, we employed several filters to extract high-quality variant sites. We removed sites found to be significantly out of Hardy-Weinberg equilibrium (*p*‐value < 0.001) and required a minimum depth of 3× using vcftools' --hwe and --minDP flags, respectively. Prior to downstream analyses, we additionally used VCFtools v0.1.15 [44] to extract autosomes and filter out sites with quality scores lower than 30, variants with greater than 10% missing genotypes and a minor allele frequency of less than 5%, retaining 3 548 561 sites. Additionally, we determined carriers of t-haplotypes, validated sex and determined relatedness (electronic supplementary material, text S2 and S3, electronic supplementary material, tables S1–S4).

### (c) Analysis of population structure and phylogenetics

For phylogenetic analyses and analyses of population structure, autosomal sites were extracted and pruned for pairwise linkage disequilibrium above 0.1 (PLINK v.1.9; −indep-pairwise 50 50 0.1) [45,46]. Following pruning, 659 303 sites were retained. Principal component analysis was carried out using PLINK for house mouse populations and smartpca for human populations [47,48]. Proportions of shared ancestry in house mice were estimated using ADMIXTURE [49]. The optimal number of distinct ancestral source populations (K) for ADMIXTURE was determined using mean 10-fold cross-validation error. Phylogenetic relationships of populations were estimated from a maximum likelihood tree constructed using RaxML [50]. The maximum likelihood tree was constructed first using RAxML under a GTRGAMMA model with bootstrapping and correcting for ascertainment bias using the Lewis model.

ADMIXTURE analyses suggested that gene flow had occurred between some populations (see §3). To further study gene flow, we used the topology inferred with RAxML as a newick population tree to compute *f*-branch statistics using D-Suite [51]. To get a better sense of the genetic similarity between any two populations, we computed outgroup *f_3_* statistics. Outgroup *f_3_* statistics measures similarity in two populations by the degree of shared genetic drift relative to an outgroup (i.e. higher *f_3_* values correspond to greater genetic similarity). Normalized *f_3_*-statistics were computed using ADMIXTOOLS qp3Pop, specifying samples from Iran as the outgroup [52]. Significance of IBD was evaluated using the Mantel test (*vegan* v2.6−4) based on 10 000 permutations and Pearson’s correlation [53,54], and associated regression lines were generated in MS Excel.

### (d) Population size and divergence times

We used smc++ (vers. 1.15.2) to estimate the history of effective population size for each European population and divergence times between each pair of populations using default parameters [55]. The inferred ancestral population sizes cover the time since house mice colonized Europe in the last several millennia as well as the time since the divergence of *M. m. domesticus* from *M. m. musculus* and *M. m. castaneus* 200 000−500 000 years ago. Simulations suggest that smc++ provides accurate estimates of population size and divergence times over this range [55]. For each population, biallelic autosomal sites were extracted using vcftools options --min-alleles 2 and --max-alleles 2. Only one individual from each set of related mice in electronic supplementary material table S3 was retained for demographic inference. *vcf2smc* was called separately for each chromosome and stored in population specific data files. For each data file, cross-validated historical population sizes were estimated using the smc++ *cv* command with the --fold option set to the number of provided population samples. Split times were inferred using the smc++ *split* command on the consensus best k-fold cross-validated parameter estimates provided by smc++ *cv* output. The pairs of populations used here correspond to well defined genetic clusters in phylogenetic and ADMIXTURE analyses of K = 9 and 10 (electronic supplementary material, figures S1 and S2). The mutation rate was set to 4 × 10^–9^ per generation per base pair, and we used a generation time of 1 year [13]. In temperate climates, most house mice breed seasonally, although some commensal populations can breed year-round depending on the availability of food. We used a conservative estimate of one generation per year based on the assumption of seasonal breeding, as done in Gray et al. [56], Phifer-Rixey et al. [18] and Fujiwara et al. [19].

## 3. Results

### (a) Genome sequences

We sequenced whole genomes of 59 mice from Italy, France, Portugal, Spain, England, Scotland, Wales and Guernsey to an average depth of 13× and aligned reads to the GRCm38/mm10 reference genome. The average depth of coverage on the autosomes was 13.41× (±2.64 s.d.), ranging from 7.22× to 19.93×, comparable to the depth of coverage found among the 24 samples published by Harr *et al*. [39], which ranged from 14× to 24×. Average per-base depth of coverage for each individual, separated by autosomes, X-chromosome and Y-chromosome, is provided in electronic supplementary material, table S1.

### (b) Analysis of population structure and phylogenetic relationships

**Figure 1.**
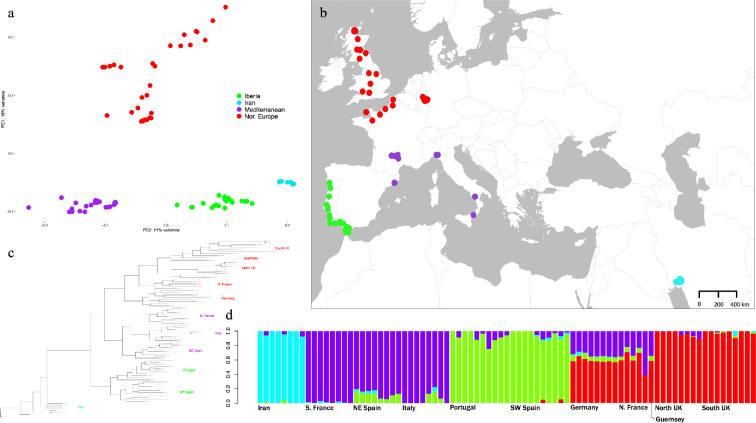
Sample distribution and population structure of western house mice. (a) Principal component analysis. (b) Sample map depicts locations (colour, year caught) in Portugal (green, 2005), southwest Spain (green, 2008), southern France (purple, 2005), Italy (purple, 1987 and 1991), northeast Spain (purple, 1991), England (red, *ca* 1990, 2001, 2004 and 2008), Wales (red, 2005), Scotland (red, 1989, 1996, 1997, 1999 and 2004), Guernsey (red, 2007), northern France (red, 2007), Germany (red, 2006) and Iran (cyan, 2006) from which western house mouse genomes were obtained or were previously available. Samples from southern France (purple), Germany (red) and Iran (cyan) are from [39]. (c) Maximum-likelihood phylogeny and (d) ADMIXTURE analysis identify four distinct groups corresponding to samples from northern Europe (red), the Mediterranean (purple), the Atlantic coast of Iberia (green) and Iran (cyan).

Principal component analysis revealed four distinct clusters (figure 1a). Western house mice from Germany, northern France, Guernsey, England, Scotland and Wales form a northern European cluster. Mice from southern France, Italy and northeast Spain form a Mediterranean cluster. We use the terms northern European and Mediterranean to be consistent with previous studies which also identified such groupings in house mice [17,31]. Mice from Portugal and southwest Spain form an Atlantic Iberian cluster. Samples from Iran form their own distinct cluster.

These population groupings are also seen in ADMIXTURE analysis. The ADMIXTURE level of K with highest support (by lowest cross-validation error) is *K* = 4 (figure 1d). At *K* = 4, we again observed a distinct cluster for Iran, the northern European samples, the Mediterranean samples and the Atlantic Iberian samples. ADMIXTURE results also suggest shared ancestral proportions between the Mediterranean group and the populations of northern France, Guernsey and Germany at multiple levels of K (electronic supplementary material, figure S1), suggesting gene flow across continental Europe from the Mediterranean to northern Europe.

Within Europe, we observed three major monophyletic groups, corresponding to the groups identified by PCA and ADMIXTURE: an Atlantic Iberian clade, a northern European clade and a Mediterranean clade (figure 1c). Samples from Iran were paraphyletic with respect to the samples from Europe. The Atlantic Iberian clade was the sister group to the other two groups. Within the Atlantic Iberian clade, samples from Portugal were monophyletic as were samples from southwest Spain. Thus, despite their evenly spaced and continuous geographic sampling (figure 1b), mice from Portugal and southern Spain did not overlap and grouped strictly by geopolitical region. Within the Mediterranean clade, mice were also grouped by country, with mice from northeastern Spain forming a clade that was the sister group to clades of mice from Italy and southern France. Finally, the northern European group consisted of clades corresponding to Germany, northern France, the UK and Guernsey. Overall, these phylogenetic relationships agree well with PCA and ADMIXTURE analyses.

In our analyses of population structure and phylogenetics, eight samples from Scotland (six from Sutherland and two from Perthshire) show greater affinity to one another than to the nine remaining UK samples (electronic supplementary material, figures S1 and S2). In subsequent analyses, we use the nine Western European clades identified in the phylogenetic tree and therefore consider the UK samples as two separate groups: a northern UK group and a southern UK group. We note that one mouse from Inverness, Scotland shows greater affinity to the eight southern UK samples though being sampled geographically closer to the northern UK samples. This may represent a recent dispersal event. The remaining eight southern UK samples come from various parts of England and Wales, and the Dumfries and East Lothian regions of Scotland, all which are located south of the northern UK samples.

### (c) Genome-wide patterns of differentiation and isolation by distance

**Figure 2.**
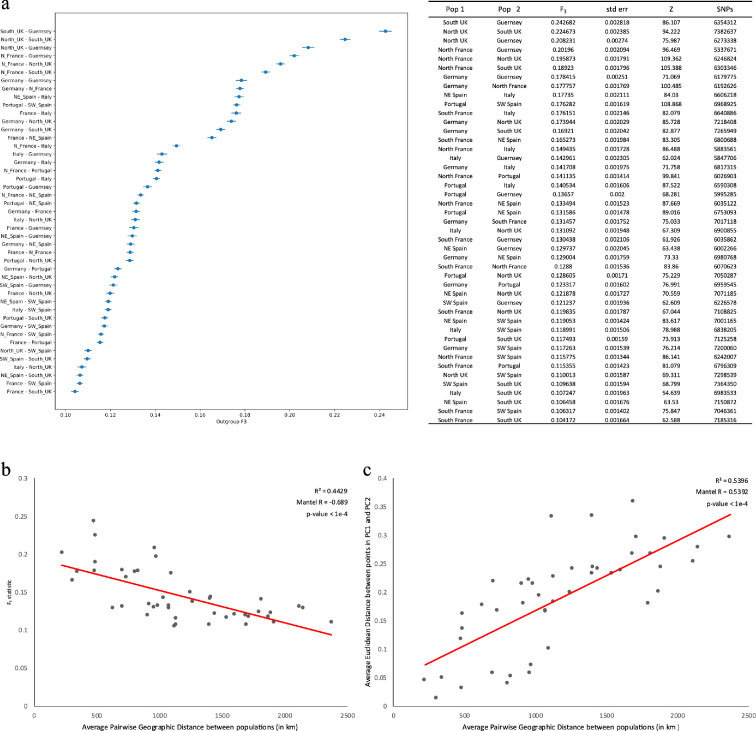
Pairwise comparisons of genetic similarity in western house mouse populations. (a) Left: biplot of normalized *f_3_* statistics. Right: table of normalized *f_3_* statistics, with z-score computed using qp3Pop. (b) *f_3_* statistics found in (a) plotted against pairwise distances between averaged geographic coordinates of each population. (c) Average Euclidean distance between points in PC1 and PC2 of principal component analysis found in figure 1*a* plotted against average pairwise distance between populations. *p*-values reported are from Mantel’s test using Pearson correlation.

Outgroup *f*_3_ statistics further corroborate the distinctiveness of the northern European, Mediterranean and Atlantic Iberian groups. The pairwise outgroup *f*_3_ statistic measures shared drift between each pair of populations relative to a designated outgroup. Higher values indicate greater genetic similarity between the two test populations. We computed the statistic for each pair of western European populations identified in the phylogenetic analysis, setting Iran as the outgroup. Eight of the 10 possible pairs of northern European populations gave the highest values for the outgroup *f*_3_ statistics (figure 2). It is interesting to note that the southern UK samples (England, Wales and southern Scotland) and the sample from Guernsey share the most genetic drift of all measured samples. Guernsey is an island in the English Channel geographically close to France but with cultural and political ties with the UK. Given that Guernsey was represented by a single individual, due to limitations of the methods used and the possibility of introducing bias from inadequate sampling, Guernsey was not included in the remaining analyses of gene flow and demographic inference.

Other intra-group pairs also generate high values for the outgroup *f*_3_ statistics such that all intra-group pairs provide the highest 14 values. The pairs of samples between different groups with high *f*_3_ statistic values were between Italy and northern France, Italy and Guernsey, and Italy and Germany. Each of the northern European populations in those pairings were previously inferred to have high levels of admixture from the Mediterranean group.

When dispersal distance is short relative to the geographic area under consideration, genetic similarity is expected to decay as a function of geographic distance [57,58]. This pattern of IBD is observed in human populations from western Europe [36]. To see whether mouse populations show patterns similar to those seen in humans, we computed pairwise geographic distance in km between pairs of populations using average longitude and latitude measurements for each population. Genetic similarity as measured by *f*_3_ decays as a function of geographic distance (figure 2b). In human populations from Europe, the first two principal components of genetic variation across the genome are correlated with longitude and latitude [36,47,59]. In mouse populations, we also found that latitude was strongly correlated with PC1 (0.82, *p*‐value = 2.37e−21), and longitude was correlated with PC2 (0.50, *p*‐value = 1.75e−6). Moreover, when comparing PC1 and PC2 together, we found that the Euclidean distance between averaged population centres in the two-dimensional PC space increases with geographic distance (figure 2c). This pattern of IBD closely mirrors patterns seen in humans (e.g. [36]; electronic supplementary material, figure S3).

### (d) Gene flow

**Figure 3.**
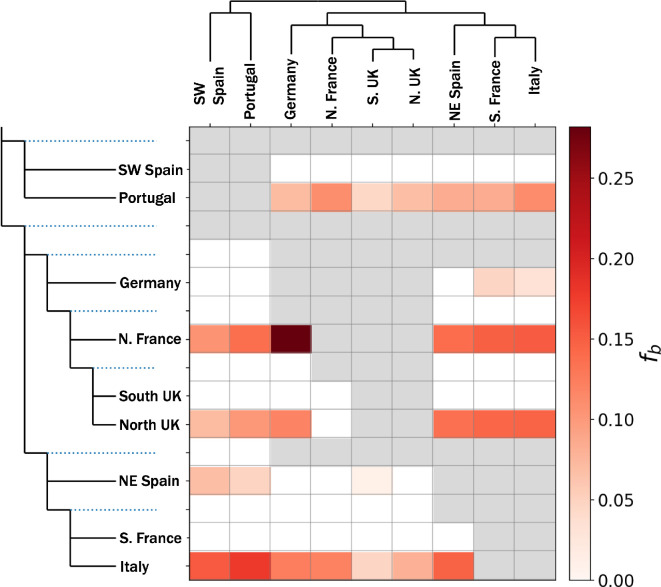
Signals of gene flow between western European house mouse populations. Coloured cells in the matrix highlight excess allele sharing between populations along the x-axis and branch along the expanded phylogeny on y-axis. Cells are shared from white (*f_b_* = 0) to dark red (highest value) according to *f_b_*, the *f*-branch statistics, as described in Malinksy et al. [51]. The program sets *f_b_* to 0 for any non-significant tests, since very short internal branches can lead to large but non-significant values even in the absence of gene flow. Grey squares correspond to cells whose value cannot be computed because of the structure of the tree. For example, there is no four-taxon test that enables comparison of the most basal internal lineages (e.g. the top row).

The *f*-branch statistic assigns gene flow to specific branches of a phylogeny based on excess allele sharing. This analysis revealed gene flow between the three major clades. The strongest signals of gene flow involved Portugal, northern France, northern UK and Italy, as depicted through the shaded red cells along the corresponding rows of figure 3. There are many possible four-taxon D tests in a phylogeny of even modest size. As a result of the phylogenetic non-independence of these tests, gene flow can introduce correlated D-statistics in related lineages [51]. For example, the horizontal red bars associated with Portugal probably reflect gene flow involving Portugal and one of the other populations in the northern European or Mediterranean clades, with the remaining red squares reflecting the correlated signal in related lineages. The single largest signal was between northern France and Germany, which are also the two populations for which ADMIXTURE analysis infers complex admixture histories. Northern France also appears to have a strong signal of gene flow with Portugal, suggesting gene flow along the Atlantic seaboard, a result consistent with previous studies [26,60–62].

### (e) Inferring past population bottlenecks

**Figure 4.**
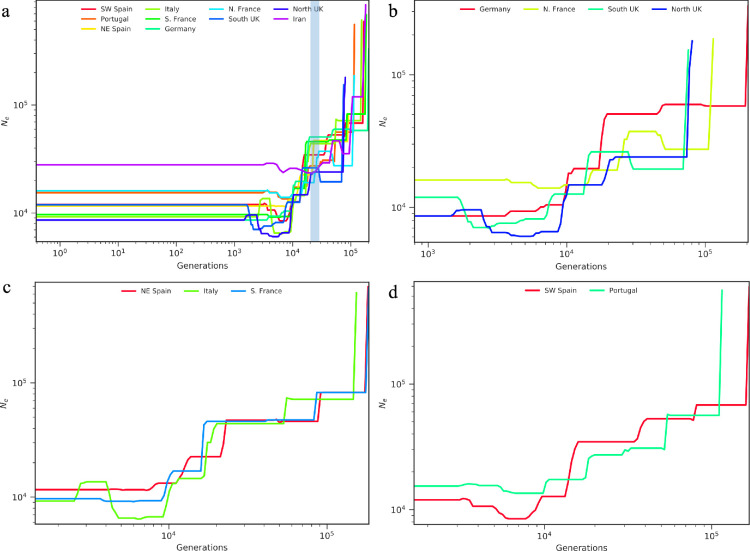
Demographic history of western house mice inferred using smc++. Plot of effective population size changes in (a) all populations (with light blue shaded region indicating the period of the Last Glacial Maximum), (b) northern European populations, (c) Mediterranean populations and (d) Iberian populations.

All populations of house mice experienced a population size decline from 500 000 to roughly 20 000 generations ago, at which point the effective population size in Iran began to stabilize (figure 4). Using a generation time of 1 year [13], 20 000 years ago coincides with the Last Glacial Maximum. After this time, the Western European populations continued to experience a reduction in population size relative to Iran. The first Western European populations began to stabilize inside of 10 000 generations ago, and all Western European populations stabilized to their present effective population size within the past 1500−3000 generations (figure 4).

### (f) Divergence times between lineages

**Figure 5.**
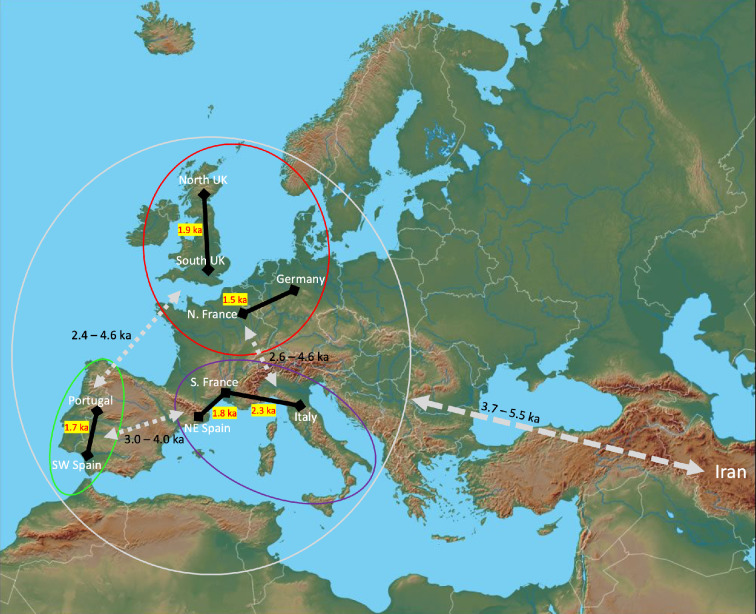
Summary of inferred divergence times of sampled house mouse populations based on smc++ (electronic supplementary material, table S5). This summary updates previous models that were based on microarrays, mtDNA, and archaeological evidence ( [9,22,23,31]). Each clade comparison found in electronic supplementary material, table S5 reports inferred split time used to construct the time intervals summarized here in black text next to the grey double-sided arrows. Intra-clade comparisons of neighbouring geographic regions connected by the black line are provided in red and highlighted in yellow.

We estimated divergence times between all pairs of populations using smc++. All estimated divergence times were between 1500 and 5500 years ago (electronic supplementary material, table S5 and figure 5). The estimated divergence times between Iran and Western European mice ranged from 3700 to 5500 years ago, consistent with previous studies which inferred Late Bronze to Iron Age colonization [9,26,28]. Of the samples included in this study, the Mediterranean clade includes mice from populations that are geographically closest to the Levant, the geographic region from where mice in Western Europe are thought to have originated [9,28]. Divergence times between Iran and the Mediterranean clade were roughly between 4200 and 4700 years ago.

Divergence times between northern Europe and Iran ranged roughly between 3700 and 5100 years ago, while divergence times between northern Europe and the other two western European clades generally ranged from 2400 to 4600 years ago. Divergence times between Atlantic Iberian mice and Mediterranean mice were estimated to be approximately between 3100 and 4000 years ago. The wider range of divergence times for comparisons involving northern European populations might reflect a more complex demographic history within the northern European clade, as analysis of population structure and *f*-statistics suggests notable admixture in northern France and Germany.

We also estimated divergence times among populations within major clades. These times ranged between 1500 and 3300 years ago. If we only consider populations that geographically border one another, these divergence times are more recent, ranging from 1500 to 2500 years ago (electronic supplementary material, tables S5 and figure S4; figure 5). The populations with the most recent split times were those in Germany and northern France (1500 years ago), followed by Spain and Portugal (1700 years ago). smc++ infers divergence times between pairs of populations under a ‘clean split’ model [55], assuming no gene flow post-split between populations. In the case of gene flow occurring following divergence, a simple split model is expected to underestimate divergence times [63].

### (g) Comparison of human and house mouse genetic distances

To quantify the concordance between patterns of genetic variation in house mice and in humans, we directly compared principal-component summaries of genetic variation genome-wide in both species. Lazaridis *et al.* [64] studied 2345 contemporary humans across Europe and the Near East who were genotyped with the Human Origins array. Their PC analysis revealed genetic clusters of individuals from Iran, the Iberian Peninsula, the Mediterranean and northern Europe, among others. Of the individuals in Lazaridis *et al*. [64], we identified populations from Iran, Italy, northeast Spain, southern France and the UK that were sampled from relatively similar locations as our mice. Among these human populations, we note that (i) Scottish and English populations cluster closely together, (ii) Spanish, French and Italian samples cluster south of the UK samples in proper west to east orientation and (iii) all of these western Europeans cluster separately west of the Iranian samples. These patterns are also reflected in the house mouse samples (electronic supplementary material, figure S3a,b). We compared the pairwise Euclidean distance between average PC1 and PC2 coordinates for each population in human and house mouse populations and found a modest but significant positive correlation (ρ = 0.574, *p*‐value = 0.01822) (electronic supplementary material, figure S3c).

## 4. Discussion

*Mus musculus domesticus* is the most widespread subspecies of *M. musculus* and can be found across the Middle East, western Europe, Africa, the Americas and Australasia. Western Europe is the historical range of *M. m. domesticus* from which mice have been introduced to other regions of the world within the past few hundred years. Understanding the demographic history of house mice within their more ancestral range provides a context for understanding their spread to other parts of the globe. Gaining an understanding of the genetic history of house mice globally therefore requires whole-genome data from a geographically diverse sampling of house mice in western Europe. For these reasons, we introduce 59 newly sequenced whole genomes of *M. m. domesticus* from western Europe. To provide a comparative context for this dataset, we combined these sequences with a previously published dataset of 24 whole genomes and analysed population structure and inferred demographic history.

Since house mice are commensal with humans, we predicted that patterns of population structure in house mice would reflect patterns previously found in humans. This appears to be largely true. For example, the sampled populations of house mice form three major clades in western Europe (figure 1). Similarly, genetic clusters of human populations from comparable regions of western Europe (i.e. the Iberian Peninsula, the Mediterranean and northern Europe) have been observed in previous studies (electronic supplementary material, figure S3) [33,34,64–67]. Though ideally our sampling could better cover gaps between genetic clusters, there are instances where geographic distance between populations within a genetic cluster (i.e. southern Italy and northeast Spain) notably exceeded the distance between the nearest populations belonging to two different genetic clusters. We found that mice from France are divided into those in the north that show clear affiliations with mice in the UK and Germany and those in the south that show clear affiliations with mice from Italy and northeastern Spain, a pattern also found in human populations from these regions [64]. We also observed a strong effect of IBD in mouse populations (figure 2), mirroring patterns seen in humans from these same regions [34,36,65,66]. Finally, we compared pairs of populations and observed a modest, but significant, positive correlation of the genetic distance in PC space for mouse and human samples (electronic supplementary material, figure S3c), indicating large scale concordance between humans and mice in the genetic structuring of populations.

On a smaller geographic scale, the genetic structure of mouse populations sometimes, but not always, matched current geopolitical boundaries. For example, the grouping of mice from the Atlantic coast of the Iberian Peninsula identified a Portuguese clade and a Spanish clade, despite the closer proximity of some Spanish mice to some Portuguese mice relative to other mice sampled within the same country (figure 1c). Mice along the Atlantic Iberian coast were sampled in an evenly spaced fashion, with the closest samples between Portugal and Spain being from Tavira (Portugal) and Aljaraque (Spain) which are only 65 km apart. Yet, an unsupervised phylogenetic reconstruction of these populations is able to clearly resolve mice from Portugal as distinct to mice from Spain (figure 1c). In other cases, mice do not match current geopolitical boundaries. For example, Spanish mice along the Atlantic Iberian coast grouped separately from mice sampled in the northeastern Catalonian region of Spain.

Inferences of migration among mouse population may also be explained by human movements. For example, studies of ancient DNA have revealed high mobility among human populations across Europe over the past 3000 years [33,34,64–68]. Leslie *et al.* [68] estimated that human populations in Britain possess substantial genetic contributions from human populations in northern France and Germany. They suggest that the historical context of this contribution centred around waves of human migration associated with the expansion of the Roman Empire. Possibly mirroring this pattern in humans, admixture analysis revealed that house mice from Germany and northern France experienced gene flow from the UK and the Mediterranean (figure 1d). Germany and northern France are separated from the UK by the English Channel and from the Mediterranean by several hundred miles. These distances and significant geographic features probably were not traversed by house mice without the assistance of humans. More generally, gene flow highlights the importance of maritime routes in the spread of house mice. Evidence of gene flow between Portugal and northern Europe supports previous reports of house mice in some islands of the North Atlantic having come from northern Europe, despite greater proximity and historical links to mainland Portugal [60–62].

Some of the patterns documented here using whole genomes provide support for previous studies which alluded to similar relationships using mitochondrial DNA. Searle *et al.* [21] and García-Rodríguez *et al.* [26] found that British populations of house mice exhibit high mitochondrial DNA variation and that northern Germany and southern and central British populations contained a different haplogroup from that seen in mice from northern Scotland and Ireland. Our analysis of population structure revealed similar patterns in the distinctness of northern and southern UK populations, reflecting patterns observed in human UK populations [34,68].

Divergence times between populations can also shed light on the joint history of humans and house mice. The estimated divergence times between geographically close populations within each of the three major clades were between 1500 and 2500 years ago. Cucchi *et al.* [28] emphasized the Iron Age as being the time of mass movement of mice by humans, but our molecular dating also suggests the most proximate divergences occurring slightly later—in the Roman period. The Roman Republic began about 2500 years ago and lasted until about 2000 years ago and was followed by the Roman Empire which ended approximately 1500 years ago [69,70]. The estimated divergence time between populations of mice in Italy and northeastern Spain (2277 years ago) and populations of mice in Italy and south France (2339 years ago) are close in dating and both precede the estimated divergence time between northeastern Spain and south France (1843 years ago). This is consistent with previous diffusion routes proposed along the northern Mediterranean [9,22,28,31], with dispersal out of Italy towards southern France and then Spain during the late Iron Age and early Roman Republic.

The inferred divergence times of mouse populations within the period of the Iron Age and Roman Republic broadly overlap with periods of human innovation, migration and settlement in western Europe. These estimated divergence times are largely consistent with zooarchaeological records [9,28], demonstrating the strength of using whole-genome sequences with modern computational inferences methods to infer demographic parameters. However, we caution that molecular dating is sensitive to assumptions of fixed model constraints, such as generation time and mutation rate, and confounding mechanisms such as gene flow following divergence and linked selection that are not accounted for by inference tools like smc++. Therefore, estimates of divergence times should be interpreted accordingly. Nonetheless, these examples illustrate how demographic patterns in house mice may reflect human populations that probably facilitated their spread, as has been previously reported with black rats [10].

In summary, we introduce a genomic dataset for house mice from regions of historical relevance that were previously not represented in public datasets. We use this dataset to update our understanding of the dispersal and contemporary population structure of house mice in western Europe. We observed that many of the patterns we found in house mice match patterns previously reported in human populations or aligned well with human history. With the sampling provided by this dataset, future researchers will be more adequately equipped to explore the global dispersal history of house mice and connect potential source populations from western Europe to other extant colonies of house mice found worldwide.

## Data Availability

Raw sequence data and associated metadata can be accessed through NCBI Sequence Read Archive (BioProject PRJNA1050608). Merged vcfs containing all sampled individuals are available on Dryad [71]. Genomic data used in this study from a previously published study [39] are available under ENA PRJEB9450. Supplementary material is available online [72].
